# Limitations of the knee society score in kinematically aligned total knee arthroplasty

**DOI:** 10.1002/jeo2.12054

**Published:** 2024-06-11

**Authors:** Alexandra Leica, Manuel‐Paul Sava, Zainab‐Aqeel Khan, Rolf W. Hügli, Michael T. Hirschmann

**Affiliations:** ^1^ Department of Orthopaedic Surgery and Traumatology Kantonsspital Baselland (Bruderholz, Liestal, Laufen) Bruderholz Switzerland; ^2^ Department of Clinical Research, Research Group Michael T. Hirschmann, Regenerative Medicine & Biomechanics University of Basel Basel Switzerland; ^3^ Department of Research AO Hospital Karachi Pakistan; ^4^ Institute of Radiology and Nuclear Medicine, Kantonsspital Baselland Bruderholz Switzerland

**Keywords:** alignment, KA, kinematic alignment, knee society score, KSS, outcomes, personalised alignment, reliability, TKA, total knee arthroplasty

## Abstract

**Purpose:**

The Knee Society Scoring System (KSS) is a frequently used outcome score which quantifies functional patients' outcomes before and after total knee arthroplasty (TKA). Several problems arise when trying to implement KSS for obtaining postoperative outcomes after more personalised aligned TKAs. Scoring for valgus femorotibial angle (FTA) intervals outside moderate ranges is often poorly explained, the specific version of KSS used for outcome collection is frequently unclear and the exact measuring methods are typically not described in the literature. The aims of this systematic review were to investigate the latest user practice, the application of KSS and its limitations after kinematically aligned (KA) TKA.

**Methods:**

A systematic literature search following PRISMA guidelines was conducted on PubMed, Embase, Medline and Scopus to identify potentially relevant articles for this review, published from the beginning of January 2013 until the end of January 2023. Broad Mesh terms such as ‘kinematic alignment’, ‘total knee arthroplasty’ and ‘knee society score’ were used for building search strategy in each database accordingly. Articles reporting postoperative values of the objective surgeon‐assessed KSS after KA TKA or KA and mechanically aligned TKA were included. For assessing included randomised control trials (RCTs), an Agency for Healthcare Research and Quality's design‐specific scale for assessing RCTs was used. The non‐RCTs were assessed by using the Joanna Briggs Institute Critical Appraisal Tool. The Ottawa–Newcastle Score system was also used. Studies were additionally evaluated for their radiological methodology by using a five‐question checklist (Radiological Assessment Qualit criteria).

**Results:**

The initial search identified 167 studies, of which 129 were considered for screening. Ten studies reporting outcomes after KA TKA did not use the objective surgeon‐assessed part of KSS for clinical outcome measurement, and 30 studies reporting outcomes after KA TKA did not use KSS at all for clinical and/or functional outcomes. From the 10 included studies, only six have used the latest KSS score (2011), the rest using its 1989 variant; and out of these six studies, only two presented values of the FTA, which is needed for calculating the KSS's ‘alignment’ subcomponent, the rest presenting hip–knee–ankle angle (HKA) values. Additionally, when converting these HKA values to FTA intervals, the authors of this systematic review found that KA TKA FTA intervals display limits, which tend to be outside the ‘well‐scored’ KSS anatomical alignment interval.

**Conclusion:**

The inconsistent and nonstandardised use of the surgeon‐assessed KSS across studies reviewed compromises assessment reliability and patient outcome scores. To enhance precision and comparability, it is crucial to standardise the KSS application, incorporating personalised alignment strategies for more accurate patient evaluations.

**Level of Evidence:**

Level III.

**Abbreviations**
AHRQ: Agency for Healthcare Research and Quality's
JBI: Joanna Briggs Institute's
KA: Kinematical alignment
KSS: Knee Society Scoring System
MA: mechanical alignment
NOS: Newcastle–Ottawa Scale
PFA: patellofemoral arthroplasty
RAQ: Radiological Assessment Quality
rTKR: revision total knee replacement
TKA: total knee arthroplasty
UKA: unicondylar knee arthroplasty

## INTRODUCTION

The Knee Society Scoring System (KSS) was introduced by The Knee Society as a rating scale, which quantifies patients' outcomes before and after total knee arthroplasty (TKA) [10]. It was devised in 1989 (marginally revised in 1993) and underwent major changes in 2011, expanding in its present form [10, 13, 21]. Currently, it is one of the most frequently used scores for knee joints [18, 23]. This is due to its mixed outcomes measures, which collect objective (based on surgeon‐collected data) and subjective outcomes (based on patient‐reported data).

In the last two decades, the spectrum of patients who undergo TKA has increased, including younger patients, who tend to be physically more active [13, 15]. As a response to this, in 2011, the ‘new’ KSS was developed to prioritise the patient's perspective in the follow‐up, thus focusing more on the patient's expectations, satisfaction and activity levels [19, 21].

Concomitantly, the preferred alignment technique in TKA has shifted from a systematic mechanical alignment (MA) towards a more personalised approach. Kinematically aligned (KA) TKA, which targets the restoration of the prearthritic limb alignment for each patient, has gained increased interest and more widespread use [8, 9, 26]. The ‘new’ KSS has tried to adapt to current TKA alignment philosophies. The accepted interval for postoperative limb alignment has been widened to 2–10° valgus (anatomical axis), and clear instructions regarding a standardised method of alignment measuring (i.e., femorotibial angle [FTA] on long‐leg standing anteroposterior radiographs) have been provided [10]. Moreover, the alignment scoring system has also changed from a negative one (deducting points for outlier values without offering points for values inside the accepted interval) [10] to a mixed type (deducting points for outlier values and offering points for values inside the accepted interval) [10, 23].

However, these changes alone may prove not to be enough, as several problems arise when trying to implement KSS for obtaining postoperative outcomes after KA TKA. First, other than 2–10° valgus, FTA intervals are poorly scored without explanation [18]. Second, in the current literature, one can seldom understand which version of KSS is used for collecting outcomes. Third, often the exact measuring method is not described. Frequently, mechanical hip–knee–ankle angle (HKA) appears to be used instead of FTA when calculating KSS's ‘alignment’ subcomponent. All of these issues may translate into a low reliability of the ‘surgeon assessed’ KSS when used for collecting and interpreting clinical outcomes after KA TKA [1].

The aims of this systematic review were to investigate the latest user practice, the application of KSS and its limitations after KA TKA. It was hypothesised that KSS is currently avoided or not implemented in a standardised manner in assessing clinical outcomes after KA TKA.

## METHODS

A systematic literature search following PRISMA guidelines was conducted on PubMed, Embase, Medline and Scopus to identify potentially relevant articles for this review, published from the beginning of January 2013 until the end of January 2023. Mesh terms such as ‘kinematic alignment’, ‘total knee arthroplasty’ and ‘knee society score’ were used for building a search strategy in each database accordingly. A detailed description of the search strategy can be found in Table 1. Identified studies have been imported into Covidence® (Veritas Health Innovation Ltd.) and removal of duplicates has been automatically performed. Two authors independently underwent title and abstract screening. Full‐text analysis has been performed by the same two authors. In case of disagreement/uncertainty, a third author was consulted. The selection was based on the following inclusion criteria: full‐text English‐ or German‐language clinical studies published in peer‐reviewed journals, which collected objective/clinical/surgeon‐assessed KSS scores following TKA. Not original research, preprints, abstract‐only studies, protocols, literature reviews, meta‐analyses, expert opinion articles, book chapters, surgical technique studies and studies pertaining to unicondylar knee arthroplasty, patellofemoral arthroplasty or revision total knee replacement, were excluded. Only articles reporting preoperative and postoperative values of objective surgeon‐assessed KSS after KA TKA or KA and MA TKA were included. Studies with unavailable numeric data (graphical only) were also excluded.

**Table 1:** Overview of selected studies.

References	Year published	Study design	Evidence level	FU (months)	No of patients	Age (years, mean/median ± SD/limits)	Gender (males, %)
Matsumoto et al. [16]	2019	Prospective cohort study	II	12	KA: 30	KA: 74.2 (55–86)	KA:16.7% (5)
					MA: 30	MA: 75.5 (61–86)	MA: 13.3% (4)
Dossett et al. [3]	2014	Randomised controlled trial	I	24	KA: 44	KA: 66 ± 7.7 (51–84)	KA: 95.3% (41)
					MA: 44	MA: 66 ± 8.6 (47–86)	MA: 88.3% (38)
Dosset el al. [4]	2012	Randomised controlled trial	I	6	KA: 41	KA: 65 ± 8.0	KA: 95% (39)
					MA: 41	MA: 66 ± 8.2	MA: 85% (35)
Tsubosaka et al. [24]	2019	Prospective cohort study	II	12	OrthoPilot: 30	OrthoPilot: 74.2 ± 9.4 (55–84)	OrthoPilot: 16.6% (5)
					iASSIST: 30	iASSIST: 75.8 ± 7.0 (61–85)	iASSIST: 26.6% (8)
Koh et al. [14]	2021	Retrospective Cohort Study	III	24	KA: 93	KA: 69.1 ± 8.1	KA: 24.7% (23)
					MA: 93	MA: 67 ± 7.4	MA: 21.5% (20)
Niki et al. [17]	2018	Experimental study	III	31.6	KA: 45	KA: 70.4	KA: 28.9% (13)
					MA: 45	MA: 72.8	MA: 22.2% (10)
Sappey‐Marinier et al. [20]	2021	Retrospective case‐control study	III	KA: 42.9 ± 3.6 (37.6–46.7)	KA: 50	MA: 70 ± 8.5 (49–86)	KA: 42% (42)
				MA: 53.3 ± 4.1 (45.5–59.8)	MA: 100	KA: 68.2 ± 8.9 (53–85)	MA: 32% (16)
Jeremić et al. [11]	2020	Retrospective case–control study	III	12	KA: 24 MA: 24	KA: 70.7 ± 6.7 (55–80)	KA: 45.83% (11)
						MA: 72.5 ± 5.8 (60–85)	MA: 45.83% (11)
Calliess et al. [2]	2016	Randomoised controlled trial	I	12	KA: 100	KA: 67 ± 8	KA: 39% (39)
					MA: 100	MA: 70 ± 8	MA: 43% (43)
Giustra F et al. [5]	2022	Retrospective case‐series	III	24	64	73.4 ± 8.2	42.2% (27)

Abbreviations: KA, kinematical alignment; MA, mechanical alignment; SD, standard deviation.

### Data extraction

**Figure 1:**
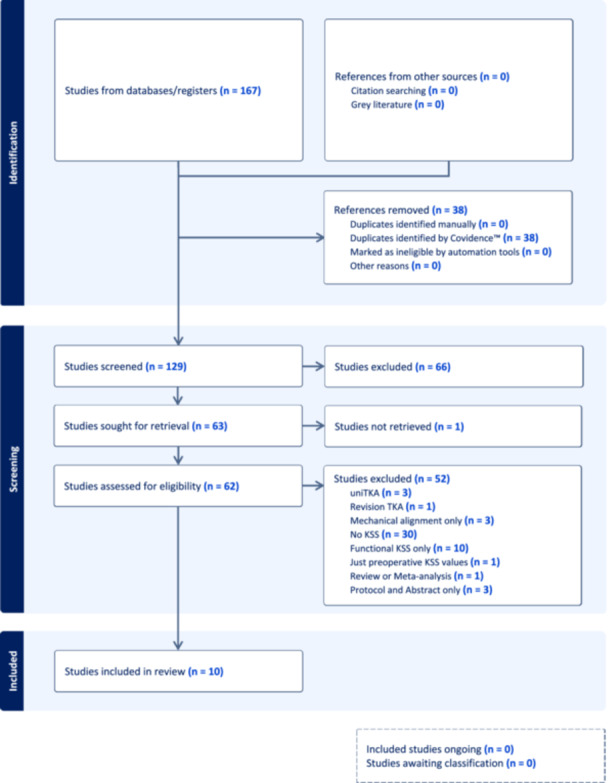
Flowchart of the study selection process according to the PRISMA 2020 statement: an updated guideline for reporting systematic reviews. KSS, Knee Society Scoring System; TKA, total knee arthroplasty.

Title, authors, year of publication, study design, level of evidence, follow‐up period, patients' demographic data, sample size, postoperative KSS scores, KSS version, postoperative alignment values, used radiological parameters, radiological protocols, statistical significance as well as other variables, were extracted for analysis. The corresponding authors of the included studies were contacted two separate times through E‐mails in case of missing data. In the event of no response, missing data has been noted with ‘nm (not mentioned)’. The flowchart of the study selection process according to the PRISMA 2020 statement is shown in Figure 1.

### Quality assessment

All included studies were assessed for their quality according to the study design. In this review, an Agency for Healthcare Research and Quality's design‐specific scale for randomised control trials (RCTs) was implemented [25] (Table 2). The non‐RCTs were assessed by using the Joanna Briggs Institute's Critical Appraisal Tool [12] (Table 3). The Newcastle–Ottawa Scale was also used to assess case–control and cohort studies [22] (Tables 4, 5). Studies were additionally evaluated for their radiological methodology by using a five‐question checklist (Radiological Assessment Quality [RAQ] criteria) [6]. Studies were categorised as low, medium or high risk of assessment bias based on the radiological methods described (Table 6).

**Table 2:** Quality assessment criteria for RCTs.

Quality assessment	Dossett et al. [3]	Dossett et al. [4]	Calliess et al. [2]
Was the allocation sequence generated adequately?	Yes	Yes	Yes
Was the allocation of treatment adequately concealed?	Yes	Yes	No
Did researchers rule out any unintended exposure that might bias results?	No	No	No
Were participants analysed within the groups they were originally assigned to?	Yes	Yes	Yes
Was the length of follow‐up different between the groups?	No	No	No
Were the outcome assessors blinded to the intervention or exposure status of participants?	Yes	Yes	No
Were the potential outcomes prespecified by the researchers? Are all prespecified outcomes reported?	Yes	Yes	Yes
If attrition was a concern, were missing data handled appropriately?	Yes	Yes	Yes
Were outcomes assessed using valid and reliable measures across all study participants?	Yes	Yes	Yes
Judgement on risk of bias?	Low risk	Low risk	Low risk

*Note*: Assessed using AHRQ design‐specific scale.

Abbreviations: AHRQ, Agency for Healthcare Research and Quality; RCT, randomised control trial.

**Table 3:** Quality assessment criteria for non‐RCTs.

Quality assessment	Niki et al. [17]	Matsumoto T et al. [16]	Tsubosaka et al. [24]
Is it clear in the study what is the ‘cause’ and what is the ‘effect’?	Yes	Yes	Yes
Were the participants included in any comparisons similar?	Yes	Yes	Yes
Were the participants included in any comparisons receiving similar treatment/care other than the exposure or intervention of interest?	Yes	N/A	N/A
Was there a control group?	Yes	Yes	Yes
Were there multiple measurements of the outcome, both pre and post‐intervention/exposure?	No	No	No
Was follow‐up complete, and if not, were differences between groups in terms of their follow‐up adequately described and analysed?	Yes	Yes	Yes
Were the outcomes of participants included in any comparisons measured in the same way?	Yes	Yes	Yes
Were outcomes measured in a reliable way?	Yes	Yes	Yes
Was appropriate statistical analysis used?	Yes	Yes	Yes

Abbreviations: JBI, Joanna Briggs Institute; RCT, randomised control trial.

*Note*: The JBI Critical Appraisal Tools for use in JBI systematic reviews.

**Table 4:** Quality assessment criteria for case–control studies.

Quality assessment	Jeremić et al. [11]	Sappey‐Marinier et al. [20]
Is the case definition adequate?	Yes	Yes
Representativeness of the cases	Yes	Yes
Selection of controls	Yes	Yes
Definition of controls	Yes	Yes
Comparability of cases and controls on the basis of the design or analysis	Yes	Yes
Ascertainment of exposure	Yes	Yes
Same method of ascertainment for cases and controls	Yes	Yes
Nonresponse rate	Yes	Yes
Total Newcastle–Ottawa scale (possible nine stars)	8	8

*Note*: Newcastle–Ottawa scale.

**Table 5:** Quality assessment of cohort studies.

Quality assessment	Koh et al. [14]	Giustra et al. [2]
Representativeness of the exposed cohort	Yes	Yes
Selection of the nonexposed cohort	Yes	Yes
Ascertainment of exposure	Yes	Yes
Demonstration that outcome of interest was not present at the start of the study	Yes	Yes
Comparability of cohorts on the basis of the design or analysis	Yes	Yes
Assessment of outcome	Yes	Yes
Was follow‐up long enough for outcomes to occur	Yes	Yes
Adequacy of follow‐up of cohorts	Yes	Yes
Total Newcastle–Ottawa scale	8	8

*Note*: Newcastle–Ottawa scale.

**Table 6:** Radiological methods: quality assessment of included studies (RAQ criteria).

References	Modality of image	Timing of image	Weight‐bearing	Protocol Standardisation	Rater reliability assessment	Outcome
Matsumoto et al. [16]	LLR, CT	12 months	N	U	Y	Medium risk
Dossett et al. [3]	AP knee radiographs, CT	24 months	Y	U	N	High risk
Dossett et al. [4]	LLR, CT	6 months	Y	Y	N	Medium risk
Tubosaka et al. [24]	LLR	1 and 12 months	Y	U	N	High risk
Koh et al. [14]	U	6 and 24 months	N	U	Y	Medium risk
Niki et al. [17]	LLR, CT	2 weeks and 31.6 months	Y	U	N	High risk
Sappey‐Marinier et al. [20]	LLR	36 months	N	U	N	High risk
Jeremić et al. [11]	LLR	12 months	Y^a^	Y^a^	U	Low risk
Calliess et al. [2]	LLR	12 months	Y	U	N	High risk
Giustra et al. [5]	LLR	24 months	Y	Y	U^a^	Low risk

*Note*: Assessment of radiological methods used to assess alignment for this review. A five‐point checklist (Figure 1) was devised, and all studies were assessed using this checklist to identify whether they were high/low risk.

Abbreviations: AP, anteroposterior; CT, computerised tomography; LLR, long leg radiograph; N, no; RAQ, Radiological Assessment Quality; U, unknown; Y, yes.

^a^ Information collected after discussing with the authors.

### Statistical analysis

Continuous variables were defined by using descriptive statistics such as means, standard deviations and percentages. The quality assessment was performed for all the content used in this systematic review. A *p* < 0.05 was considered statistically significant.

## RESULTS

### Search results

The initial search identified 167 studies, of which 129 were considered for screening. Ten studies reporting outcomes after KA TKA did not use the objective surgeon‐assessed part of KSS for clinical outcome measurement. Thirty studies reporting outcomes after KA TKA did not use KSS at all for clinical and/or functional outcomes. Details of the study selection and inclusion process are illustrated in Figure 1 (PRISMA flowchart). Ten studies completely fulfilled the inclusion criteria [2, 3, 4, 5, 11, 14, 16, 17, 20, 24], including three RCTs [2, 3, 4], three case series [16, 17, 24], two case–control studies [11, 20] and two retrospective cohort studies [5, 14].

Eight studies compared postoperative values for the objective surgeon‐assessed KSS between MA TKA and KA TKA [2, 3, 4, 11, 14, 16, 17, 20], and two reported these values for patients after KA TKA only [5, 24]. The total number of patients recruited for KA TKA throughout the included studies was 551. Patients' baseline characteristics were comparable between studies (Table 1).

### Quality assessment of the imaging methods

The radiological characteristics of each study using the RAQ criteria [6] are presented in Table 6. Different imaging methods have been reported for measuring postoperative alignment: long‐leg radiographs and computed tomography [2, 3, 4, 5, 11, 16, 17, 20, 24]. One included study did not clearly mention the imaging method [14]. Five studies [2, 5, 11, 17, 24] have reported the use of weight bearing, while five studies [3, 4, 14, 16, 20] did not mention it. Two studies [14, 16] reported the inter‐ and/or intrarater reliability for radiological alignment measurements. The corresponding authors of the other two studies confirmed, when contacted via E‐mail, that inter‐ and/or intra‐rater reliability analysis was carried out but was not reported [5, 11]. Six studies [2, 3, 4, 17, 20, 24] did not report inter‐ and/or intrarater reliability, and the corresponding authors did not reply to the sent E‐mails. The quality of the radiological assessment method RAQ criteria showed, that just two studies [5, 11] were deemed low risk of radiological assessment bias, three studies [4, 14, 16] were classified as medium risk and the remaining five studies [2, 3, 17, 20, 24] as high risk (Table 6).

### Version of used KSS and postoperative KSS scores

Four studies used the 1989 version of KSS for analysing postoperative clinical outcomes at six months, 1 and 2 years after KA and MA TKA [2, 3, 4, 14]. With the exception of Callies et al. [2], who reported just combined KSS values (clinical and functional), the rest of the three studies reported superior surgeon‐assessed KSS in the case of KA TKA, when compared with MA TKA. However, none of these findings qualified as statistically significant (*p* = n.s.). Six studies have used the 2011 version of KSS for analysing postoperative clinical outcomes at 1, 2 and 4 years after KA and MA TKA [5, 11, 16, 20, 24]. Except for Sappey‐Marinier et al. [20], which reported just combined KSS values, the rest of the five studies reported surgeon‐assessed KSS separately [5, 11, 16, 24]. The majority of these studies reported higher surgeon‐assessed KSS in the case of KA TKA when compared to MA TKA. However, with the exception of Jeremić et al. [11] (*p* = 0.02), none of these studies state their findings as statistically significant (*p* = n.s). Detailed data can be found in Table 7.

**Table 7:** Postoperative clinical/objective/surgeon‐assessed KSS scores, HKA, FTA and the corresponding KSS ‘alignment’ score values.

References	KSS (version)	KSS objective postoperative values	*p* Value	HKA postoperative	*p* Value	Corresponding KSS ‘alignment’ score intervals calculated with HKA (pt)	Estimated postoperative FTA interval	Corresponding KSS ‘alignment’ score intervals (pt)
Matsumoto et al. [16]	2011	KA: 95.0 ± 4.2 (85–100) MA: 92.8 ± 4.3 (84–100)	ns	KA: 181.9° ± 2° MA: 180.6° ± 2.4°	<0.001	KA: (−10)/25 MA: (−10)/25	KA: 186.9° ± 2° MA: 185.6 ± 2.4	KA: 25 MA: 25
Dossett et al. [3]	1989	KA: 84 ± 17.1 (43–100) MA: 72 ± 21.2 (25–100)	nm	KA: 180.1° ± 2.8° MA: 179.9° ± 2.5°	ns	KA: (−20)–(−9) MA: (−20)–(−9)	KA: 185.1° ± 2.8° MA: 184.9° ± 2.5°	KA: (−9)–0 MA: (−9)–0
Dossett et al. [4]	1989	KA: 90 ± 14.3 MA: 79 ± 18.2	nm	KA: 180.3° ± 2.8° MA: 180° ± 2.2°	ns	KA: (−20)–(−6) MA: (−20)–(−9)	KA: 185.3° ± 2.8° MA: 185° ± 2.2°	KA: (−9)–0 MA: (−9)–0
Tsubosaka et al. [24]	2011	KA: 94.2 ± 5 (79–100)	ns	KA: 181.7° ± 2°	ns	KA: (−10)/25	KA: 186.7° ± 2°	KA: 25
Koh et al. [14]	1989	KA 6 m: 87 ± 11.3 KA 24 m: 88.5 ± 10.7 MA 6 m: 87.1 ± 9.7 MA 24 m: 90.2 ± 7.4	ns	nm	nm	‐	‐	‐
Niki et al. [17]	2011	KA: 73.2 ± 6.5 MA: 73.0 ± 8.0	ns	nm	nm	‐	‐	‐
Sappey‐Marinier et al. [20]	2011	KA: 173.2 ± 19.6 (121–206) MA: 179.6 ± 19.6 (93–221)^a^	ns	nm	ns	‐	KA: 176.7° ± 3.6°^b^ MA: 175.8° ± 2.3°^b^	KA: −10 MA: −10
Jeremić et al. [11]	2011	KA: 94.0 (41–100) MA: 75.0 (30–95)	0.02	KA: 179.8° ± 3.6° MA: 179.7° ± 2.7°	ns	KA: (−10)/25 MA: (−10)/25	KA: 184.8° ± 3.6° MA: 184.7° ± 2.7°	KA: (−10)/25 MA: 25
Calliess et al. [2]	1989	KA: 190 ± 18 MA: 178 ± 17^a^	nm	KA: 179° ± 3° MA: 181° ± 1°	nm	KA: (−20)–(−9) MA: (−15)–(−9)	KA: 184° ± 3° MA: 186° ± 1°	KA: (−12)–0 MA: 0
Giustra F et al. [5]	2011	KA: 94.5 ± 7.7	ns	KA: 176.2° ± 3.9°	ns	KA: −10	KA: 181.2° ± 3.9°	KA: (−10)/25

*Note*: These data are presented as mean ± SD (range).

Abbreviations: FTA, femoral tibial angle; HKA, hip–knee–ankle angle; KA, kinematical alignment; KSS, Knee Society Score; MA, mechanical alignment; nm, not mentioned; ns, not significant; pt, points.

a Values from combined KSS.

b Values provided by the authors.

### Association between used KSS scores and postoperative alignment

With the notable exception of Sappey‐Marinier et al. [20] and Jeremić et al. [11], the majority of included studies reported just the mechanical HKA. For the calculation of the ‘alignment’ component score in KSS 1989/2011, the user manual [23] specifies that the FTA, measured on a long‐leg weight‐bearing radiograph, should be used. Therefore, starting from the stated postoperative mean HKA values, postoperative FTA mean value intervals have been calculated for each included study by the authors of this systematic review. The combined mean value for FTA in MA TKA in studies which used KSS 1989 was 185.3° ± 1.9° [2, 3, 4, 14]. In the case of FTA in KA TKA, which used KSS 1989, the combined mean value was 184.8° ± 2.8° [2, 3, 4, 14]. The combined FTA mean value in studies which used KSS 2011 after MA TKA was 182° ± 2.4° [11, 16, 17, 20] and 183.2° ± 3° after KA TKA [5, 11, 16, 17, 20, 24]. Several studies report superior objective surgeon‐assessed KSS scores after KA TKA when compared to MA TKA, although estimated postoperative FTA intervals and their corresponding scoring intervals present a different picture. The discrepancy becomes even more evident in studies, in which it is obvious that the used version of KSS is 1989, which, in theory, should clearly disadvantage the KA technique [2, 3, 4, 14]. Excluded from this analysis were Koh et al. [14] and Niki et al. [17], as they have provided no postoperative alignment parameter values (HKA or FTA). A comprehensive overview of mean postoperative HKA values, mean FTA intervals, as well as their corresponding KSS ‘alignment’ scores can be found in Table 7. Added for more clarity, clear corresponding KSS 1989 and 2011 alignment scores for each of the most common limb phenotypes have been presented in Table 8.

**Table 8:** Corresponding KSS 1989 and 2011 alignment scores for each of the most common limb phenotypes.

Limb phenotype based on HKA [7]	HKA Average value and the interval of limb phenotype	Estimated FTA^a^	KSS 2011 score (pt)	KSS 1989 score (pt)
VAR_HKA_6° (8.4% of population)	174° (172.5–175.5°)	177.5–180.5°	−10	(−20)–(−15)
VAR_HKA_3° (24.1% of population)	177° (175.5–178.5°)	180.5–183.5°	(−10)/25	(−15)–(−6)
NEU_HKA_0° (40.9% of population)	180° (178.5–181.5°)	183.5–186.5°	25	(−6)–0
VAL_HKA_3° (22.7% of population)	183° (181.5–184.5°)	186.5–189.5°	25	0
VAL_HKA_6° (3.9% of population)	186° (184.5–187.5°)	189.5–192.5°	(−10)/25	(−6)–0

Abbreviations: FTA, femoral tibial angle; HKA, hip–knee–ankle angle; KSS, Knee Society Score; NEU, neutral; pt, points; VAL, valgus; VAR, varus.

a The FTA is estimated by adding 5° to the given HKA.

## DISCUSSION

The most important finding of this systematic review is that although KSS is not ideal for patients who underwent KA TKA, due to the current ambiguity in the ‘alignment’ subcomponent, 10 out of 50 studies used it anyway. Within these 10 studies, only six have used the latest KSS score (2011), the rest were using its 1989 variant; and out of these six studies, just two have presented the values of the FTA, which is needed for calculating the KSS's ‘alignment’ subcomponent. Interestingly, the KA TKA scores were similar to MA TKA scores, which should not be the case, given the fact that when converting the reported HKA values to FTA intervals, the authors of this systematic review found that KA TKA FTA intervals display limits, which tend to be more often outside the ‘well‐scored’ KSS anatomical alignment interval than the MA TKA FTA. The differences in scoring can go as high as 20 points in KSS 1989 and even as high as 35 points in KSS 2011 (Table 8).

This might be due to several reasons. One of these reasons might be the high heterogeneity displayed among the studies when discussing the imaging method and protocol used for image acquisition. Another reason might be the use of a different variable (HKA) than the recommended one (FTA) in calculating the ‘alignment’ subcomponent.

There are certain limitations to this systematic review. First, the included studies displayed a high degree of heterogeneity in methodology, study design and implant choice. This was deliberate so as to include all studies relevant to the clinical application of KSS. Therefore, a number of retrospective studies with weak study design and methodology were included. Finally, there was no uniformity and clear explanation for the measurement of objective KSS in some of the studies. Therefore, it was difficult to conclude the exact mean FTA values. Calculating the FTA from a given HKA value comes with an inherent 2° error interval (FTA being 5–7° valgus to HKA).

The KSS is a widely accepted tool for outcome measurement after TKA. However, despite clear instructions in the KSS 2011 manual, there is still ambiguity in reporting and measuring the KSS objective section. This ambiguity can also be linked to the now apparent overdue update, which the KSS ‘alignment’ score component requires, in order to account for more personalised alignment techniques. Due to this ambiguity, it is difficult to conclude accurate results, especially after KA TKA, with KSS. As a result, the already observable trend of avoiding the use of KSS after KA TKA might culminate with the discontinuation of the regular use of one of the only existing clinical outcomes assessment tools after TKA.

## CONCLUSION

The inconsistent and nonstandardised use of the surgeon‐assessed KSS across studies reviewed compromises assessment reliability and patient outcome scores. To enhance precision and comparability, it is crucial to standardise the KSS application, incorporating personalised alignment strategies for more accurate patient evaluations.

## AUTHOR CONTRIBUTIONS

All authors contributed to the study's conception and design. Material preparation, data collection and analysis were performed by Alexandra Leica, Manuel‐Paul Sava and Zainab‐Aqeel Khan. The first draft of the manuscript was written by Alexandra Leica, Manuel‐Paul Sava and Zainab‐Aqeel Khan and all authors commented on previous versions of the manuscript. All authors read and approved the final manuscript.

## CONFLICT OF INTEREST STATEMENT

The authors declare no conflict of interest.

## ETHICS STATEMENT

The authors have nothing to report.
